# Who walks into vape shops in Southern California?: a naturalistic observation of customers

**DOI:** 10.1186/s12971-016-0082-y

**Published:** 2016-05-26

**Authors:** Steve Sussman, Jon-Patrick Allem, Jocelyn Garcia, Jennifer B. Unger, Tess Boley Cruz, Robert Garcia, Lourdes Baezconde-Garbanati

**Affiliations:** Department of Preventive Medicine, University of Southern California, 2001 N. Soto Street, SSB 302A, Los Angeles, CA 90032 USA; Department of Psychology, University of Southern California, Los Angeles, CA USA; Department of Sociology, University of Southern California, Los Angeles, CA USA; University of Southern California, School of Social Work, Los Angeles, CA USA

**Keywords:** Vape shop, Electronic cigarette, E-cigarette, Retail, Tobacco regulation

## Abstract

**Background:**

The rising popularity of electronic cigarettes (e-cigarettes) has been accompanied by the proliferation of vape shops in the United States. Vape shops are devoted to the sale of e-cigarettes and e-juices. This study aimed to describe the age, gender, and ethnicity of customers who frequent these shops, determine whether conversations transpire between retailers and customers, as well as identify the types of activities taking place while customers are inside the store.

**Methods:**

A naturalistic observation study of 186 customers in 59 vape shops in Southern California was completed in locations that were relatively high in Korean, Non-Hispanic white, Hispanic, or African American ethnicity.

**Results:**

Across all shops, the average estimated age of customers was 30.29 years old (SD = 9.70), 53 % were estimated to be non-Hispanic white, and 79 % were males; few minors entered the shops. Conversations about vaping related topics were prevalent (e.g., sampling e-juices, receiving help on hardware, and talking about vaping). Purchases were commonly observed as well as customers lounging in the shop.

**Conclusion:**

Vape shops provide consumers a place to purchase and discuss e-cigarettes and offer an environment that serves as a place of recreation with customers lounging once inside. Findings should inform local tobacco control efforts and regulatory policies in the future.

## Background

The recent and rising popularity of electronic cigarettes (e-cigarettes) has been accompanied by the proliferation of vape shops in the United States (U.S.) and elsewhere. Vape shops are devoted to the sale of e-cigarettes, providing a setting for customers to learn about different hardware from retailers, sample flavors, and socialize [1, 2]. Vape shops are regulated primarily at the local level, though it is illegal to sell e-cigarettes to minors (e.g., persons under 18) in various states in the U.S. including California. Currently, customers are allowed to vape in most shops, and many shops hold events such as cloud chasing competitions (e.g., blowing the largest, or longest, most dense cloud of vapor/aerosol) [3]. Research on vape shops is lagging behind their popularity [3], with initial research focusing on the marketing strategies of vape shop retailers [4], and retailers’ tobacco related attitudes and behaviors [5].

What is missing from this literature is a description of the customers who frequent these shops (e.g., age, gender, ethnicity), if conversations transpire between retailers and customers (e.g., if retailers are promoting cessation of combustible tobacco), as well as a description of types of activities that take place while customers are inside the store (e.g., if customers treat the shop as a place of recreation). The present study aimed to document vape shops in Southern California. Because tobacco-related behaviors vary by ethnicity [6], the present study engaged in a naturalistic observation of vape shops located in four different types of ethnic neighborhoods/locations (Korean, Hispanic, African American, and non-Hispanic White).

## Methods

Naturalistic observation, developed in ethology, zoology, and anthropology [7], has been utilized to investigate tobacco-related behavior among youth at retail stores and on school grounds [8, 9] and among adults while playing professional baseball [10]. This approach to data collection involves recording events in natural settings as they occur without participating in the events, use of impartial observers, and descriptions of behaviors and events which generally are well operationalized [11]. This approach was utilized to observe customers’ behavior in vape shops.

### Store selection

The locations (communities) of interest (Korean, African American, Latino, and non-Hispanic White) were selected because each of these racial/ethnic groups has a unique tobacco use profile [2], and together reflect the diversity of Southern California. Initially 104 shops were in the geographic sampling area of the study (i.e., of neighborhoods). The sampling area was identified by using published data that described the percentages by ethnicity of the Los Angeles Basin, and through the use of Yelp to locate vape shops in areas with a relatively high proportion of people from each of the four ethnicities. Yelp is a multinational corporation headquartered in San Francisco, which operates in large part as a business review site, within which customers may exchange business recommendations. The reviews involve a search engine that can locate specific types of businesses by locations/cities. Reviews permit updateable narrative description, and include a rating system (one to five stars, least to most favorable; see [2] for more details). Of the 104 identified shops, 17 were found to be out of business at the time of the interview, and 4 declined to be a part of the study. Of the 83 shops to be surveyed, 1 shop was a distributor and 5 were smoke shops (shops that sell tobacco and tobacco related products, as well as e-cigarettes) and not vape shops. The final sample included 77 vape shops (22 in non-Hispanic White neighborhood areas, 18 in Korean neighborhood areas, 17 in Hispanic/Latino neighborhood areas, and 20 in African-American neighborhood areas) in Greater Los Angeles.

### Observation form

Observation forms were used to code the approximate age of customers, gender, ethnicity, purchases made, and activities and conversations that took place. There was room to code two customers on each page of the form, and all customers observed in the shops during the data collection period were coded.

### Age estimation

To ensure that estimates regarding age were reliable, the customer observer who visited the 77 vape shops engaged in an age estimation validation pilot study [8], prior to doing the vape shop observations. The observer (a 25-year old Asian American female staff person) estimated the ages of 20 people unknown to her at a local community college campus. Among the people observed in that pilot study, half were male, with age and ethnicity varying. After writing down an estimate of each person’s age, the observer approached each person, told them that she was estimating age as part of a university study, and asked them to write down their age on a piece of paper for validation. Of the 30 people approached, 20 agreed to provide their ages. The correlation between the estimated ages (mean = 30.7, SD = 11.93, range = 7 to 63 years) and the actual ages (mean = 31.4, SD = 13.38, range = 8 to 65 years) was *r* = .95, indicating high accuracy of the estimates made. These results converge with a previous age estimation study [8].

### Procedure

Retailers were approached by data collectors between 10:00 am and 5:00 pm during workdays and were asked to participate in the study in return for a $50 gift certificate. A brief anonymous interview was conducted to determine tobacco-related behaviors and document the attitudes and beliefs of retailers towards e-cigarettes relative to other tobacco products [5]. While one data collector conducted the interview, another conducted an observation of any customers over a 20-min period. Each shop was observed one time. The observer stood at least 15 ft away from the customers, observed store products, and observed customers unobtrusively. To our knowledge customers did not know they were being observed. A “customer” was defined as any non-employee that was present inside the vape shop during the 20-min observational period. This could include a person making purchases along with anyone accompanying the purchaser. Data collection occurred from June 19, 2014 to December 8, 2014. The authors’ institution’s Institutional Review Board approved all procedures.

Time of day was not recorded. However, time of day data was collected one year later at 61 of these same shops (*n* = 225 customers). We engaged in a sensitivity analysis, conducted on that latter collected data to determine if specific observations were dependent on time of day. Vape shops were again visited between 10:00 am and 5:00 pm during workdays. About 7 % of visits occurred in the morning (10:00 am to 10:59 am), 37 % occurred in mid-day (11 am to 12:59 pm), 28 % occurred in early afternoon (1 pm to 2:29 pm), 19 % in mid-afternoon (2:30 pm to 4 pm) and 8 % in late afternoon (after 4:00 pm). The most important variable perhaps, age, failed to vary as a function of time of day of observation (F_(4, 220)_ = 1.04, *p* < .39). Observing conversations in the shops were found to vary with time of day (LR *χ*^2^_(4)_ = 14.33, *p* < .01), with conversations less likely to occur in the afternoon (after 1:00 pm) when compared to the morning (before 11:00 am). Observing purchases in the shop were found to vary with time of day (LR *χ*^2^_(4)_ = 22.61, *p* < .01), with purchases less likely to occur in the late afternoon (after 4:00 pm) when compared to the morning). Gender failed to vary with time of day (LR *χ*^2^_(4)_ = 1.90, *p* < .75) but ethnic background of customers was observed to vary (*χ*^2^_(16)_ = 32.88, *p* < .01). Non-Hispanic whites were more likely to be observed being present in shops in the morning than any other ethnic group.

### Analysis

We tabulated the frequencies of customers’ characteristics, and where appropriate, we reported means and standard deviations. Observations of customers’ characteristics and activities observed in vape shops were compared across ethnic neighborhoods by using ANOVA and chi-square tests.

## Results

### Number of customers and gender

Among the 77 vape shops in which the observation protocol was conducted, customers were observed in 59 of the shops and 186 customers were observed in total (24 % were female overall). Shops in which no customers were observed were approximately evenly distributed across ethnic location. Of 18 total Korean location shops, no customers were observed at five shops. Of 22 non-Hispanic white location shops, no customers were observed at five shops. Of 17 Hispanic location shops, no customers were observed at four shops. Finally, at 20 African American location shops, no customers were observed at four shops. Of these 59 retained shops, 51 customers were observed at 13 vape shops in Korean neighborhoods (an average of 3.92; 23 % female), 51 customers were observed at 17 shops in non-Hispanic White neighborhoods (an average 3 per shop; 29 % female), 27 customers were observed at 13 shops in Hispanic neighborhoods (an average of 2.07 per shop; 11 % female), and 57 customers were observed at 16 shops in African American neighborhoods (an average of 3.56 per shop; 16 % female). Gender failed to vary as a function of ethnic location (*χ*^2^_(3)_ = 4.90, *p* < .18).

### Age

An estimate was made on the perceived lowest and highest age of customers. The range of lowest to highest estimate was small (an average of 2.27; no more than 3 years). Across all shops, the average estimated age of customers was 30.29 years old (SD = 9.70). The average estimated age of the customers was 28.51 (SD = 10.34) in Korean location shops, 32.62 (SD = 9.22) in non-Hispanic white location shops, 30.56 (SD = 9.47) in Hispanic location shops, and 29.69 (SD = 9.47) in African American location shops. Six of 186 customers were estimated to be minors (3 %; i.e., 6, 7, 11, 14, 15, and 17 years old). In addition, six customers were estimated at a lower age range of 18. Thus, taking a conservative estimate, 12 customers possibly were minors (7 %; 8 were Asian, 3 were Hispanic, and one was non-Hispanic White). They were not involved in purchases but walked in with adults who made purchases. Six of the potential minors were in Korean location shops, and the other six were in African American location shops (in each location, half being clearly under 18 years of age). The average estimated age of the customers failed to vary by ethnic location (F_(3,182)_ = 1.65, *p* < .18).

### Ethnicity

An agreement check on ethnicity was conducted on 41 of the customers by the customer observer and one other staff person (a 25-year old Hispanic female staff person). There were only three disagreements on ethnicity among the customers (93 % agreement; the only disagreements were on three cases in which one rater thought the customer was Asian while the other rater thought the customer was non-Hispanic White). Also, the raters could not identify specific Asian ethnicity unless the customer spoke in their language of origin; thus, a general “Asian” description was coded. Among all customers observed, 21 % were Asian, 7 % were African American, 16 % were Hispanic, 53 % were non-Hispanic white, and 3 % were other. Customer ethnicity by neighborhood is described in Table 1.

**Table 1:** Vape shop customer ethnicity by ethnic location of shop

	Korean	Non-Hispanic White	Hispanic/Latino	African American
Korean/Asian	8 (16)	6 (12)	7 (26)	18 (32)
Non-Hispanic white	31 (61)	37 (73)	10 (37)	21 (37)
Hispanic/Latino	9 (18)	3 (6)	9 (33)	10 (18)
African American	2 (4)	4 (8)	0	7 (12)
Other	1 (2)	1 (2)	1 (4)	1 (2)
Total	51	51	27	57

Notes. Numbers in cells represent frequencies with percentages in parentheses. While cell sizes were too small to conduct meaningful statistical tests, the pattern of the data indicates a tendency for ethnicity of customer-ethnic location of shop concordance, except for the Korean/Asian customer ethnicity-ethnic location

### Purchases

Purchases were observed among 73 (40 %) of the customers. Twenty-one purchases were made in Korean location shops, 22 purchases were made in non-Hispanic White location shops, 10 purchases were made in Hispanic location shops, and 20 purchases were made in African American location shops. The proportion of purchases observed failed to vary by ethnic location (LR *χ*^2^_(3)_ = .87, *p* < .83). The types of purchases were noted for descriptive purposes and included e-juices, hardware such as modifiable e-cigarette systems or “mods”, vape pens, re-build payments (e.g., using new kanthal [heating] wire and a cotton wick to recoil the inside of an atomizer, which may involve the use of a drill press, replacement parts including wires, wicks, or coils, batteries or chargers).

### Conversations/Lounging

Among the customers observed, 62 % had conversations with vape shop employees while in the store. Presence of conversations varied as a function of shop ethnic location (LR *χ*^2^_(3)_ = 13.42, *p* < .01) with 43 % of customers in Korean location shops having conversations, 81 % in Hispanic location shops, 68 % in African American locations and 65 % in non-Hispanic white location shops. Types of conversations were noted for descriptive purposes and included small talk (e.g., asking how someone’s day was going), cloud chasing (e.g., discussions about competitions to determine who can blow the largest cloud of aerosol), products, service requests (e.g., repairs, rebuilds, or help with hardware), and e-juices. Observations about combustible cigarette use were not observed. Among the customers observed, 22 % were observed lounging (e.g., sitting in chairs or couches, watching TV, playing video or board games, or conversing) while in the vape shop. Lounging behavior varied by neighborhood (LR *χ*^2^_(3)_ = 13.42, *p* < .01) with 14 % of customers lounging in Korean location shops, 18 % in Hispanic location shops, in 39 % in African American location shops and 12 % in non-Hispanic white location shops.

## Discussion

The majority of customers frequenting vape shops across the four ethnic locations in Southern California were non-Hispanic white males around 30 years of age. This finding was unexpected, given the variety of neighborhoods in the sampling frame of the study. Non-Hispanic White customers were over-represented in the three ethnic minority neighborhoods. This finding suggests that non-Hispanic white males are more attracted to vape shops and their products than people from other ethnicities and females, or are more willing or able to spend disposable income on vape shop products.

Few individuals observed in the shops were estimated to be minors and when minors were observed they were accompanied by an adult and did not make purchases. With the growing use of e-cigarettes among adolescents [12], information is needed on how minors are acquiring such products. If minors are not purchasing e-cigarettes in brick and mortar retailers, they may seek out these products online [13] as a way of circumventing regulation [14]. The ability of some minors to enter these shops may expose them to nicotine-containing vapor/aerosol, possibly placing them at risk for adverse for health consequences [15, 16].

Conversation between retailers and customers was observed; however, there was little evidence of discussion about combustible cigarette smoking cessation. This finding is in contrast to other research on vape shops where retailers were observed suggesting to their patrons that e-cigarettes could be used for cessation [17], or that retailers had reported quitting conventional smoking by using e-cigarettes [5]. It would appear based on the present study that the vape shop does involve a “vape scene” (hobby/recreational activity) involving often non-Hispanic White adult males who converse about cloud chasing contests or new vaping devices, although most customers purchase products and leave.

There are several limitations of this study. For example, a 20-min block of observation time was involved and observations were made between 10:00 am and 5:00 pm Monday through Friday, a period which varied by shop. The vape shops are open on weekends. However, we were restricted by university employee policy to keep our observations on workdays. Thus, much customer activity may have been missed [18]. Sensitivity analyses of observations at these shops one year later revealed that specific observations (purchases, conversations) were more likely to occur in the morning than afternoon. Minors might be observed in the shops later in the day, although our observations did occur in several shops during a time period over which many minors might have the opportunity to enter (from 3:00 pm to 5:00 pm, right after school), and we failed to detect variation in age of customer as a function of time of day in the subsequent set of observations one year later. Customers were not approached to validate observations of gender, age, or ethnicity, due to Institutional Review Board (IRB) restrictions. However, a validation study was conducted, with results indicating that estimates of age likely were accurate [8]. Observations may have been improved if newer methods of naturalistic observation were utilized (e.g., electronically activated recorder [EAR] methodology; [19]). Methods such as EAR were not used due to IRB restrictions, and reluctance of vape shop retailers to approve the use of digitally recording events in their shops. Nevertheless, manually coding of ongoing events is a workable means to unobtrusively observe behavior as it occurs [20]. Another limitation is that only vape shops in Southern California were observed. In future research, it would be interesting to compare observations across different regions in the U.S. and perhaps different countries around the world.

These observations were conducted prior occurrence of many events in California that may have influenced e-cigarette attitudes and behaviors including a statewide anti-e-cigarette media campaign (“Wake Up”; see [21];), [16] and proposed legislation that would include e-cigarettes in indoor air provision laws (Senate Bill 140/SB 2X-5). In some locations in Southern California vape shops are now regulated in the same manner as tobacco shops. That is, the definition of smoking has been extended to include the use of e-cigarettes preventing customers from vaping and sampling juices inside the store. Future research should involve similar naturalistic observations to assess conversations or activities taking place in vape shops in response to new proposed regulations (e.g., the deeming rule), that should be considered when evaluating vendor or marketing influences on use of alternative tobacco products, and to assess potential customer health risk exposure.
